# Phenology of nesting marine turtles in the Cayman Islands

**DOI:** 10.1371/journal.pone.0338445

**Published:** 2025-12-31

**Authors:** Liliana P. Colman, Jane L. Hardwick, Timothy J. Austin, Janice M. Blumenthal, Gina Ebanks-Petrie, Brendan J. Godley, Lorri D. Lamb, Alejandro Prat-Varela, Joseph Roche-Chaloner, Annette C. Broderick

**Affiliations:** 1 Centre for Ecology and Conservation, Faculty of Environment, Science and Economy, University of Exeter, Penryn Campus, Penryn, Cornwall, United Kingdom; 2 Marine Resources Unit, Department of Environment, Grand Cayman, Cayman Islands; 3 Sharjah Centre for Marine Research, University of Khorfakkan, Sharjah, United Arab Emirates; U.S. Geological Survey, UNITED STATES OF AMERICA

## Abstract

Climate-driven shifts in the phenology of species are altering ecosystems worldwide and have been documented in many species, including marine turtles. We present a 26 year analysis of population trends and nesting phenology for green (*Chelonia mydas*) and loggerhead (*Caretta caretta*) turtles in the Cayman Islands and show that although the onset of nesting has advanced for both species (by 0.6 days.yr^-1^ for green turtles and 0.7 days.yr^-1^ for loggerhead turtles), the peak of nesting has not significantly changed. The end of the nesting season for green turtles has been delayed by 1.0 days.yr^-1^, extending the nesting season by 1.6 days.yr^-1^, whereas no significant change in season duration was observed for loggerhead turtles. Over the study period, sea surface temperature (SST) at the nesting beach has increased significantly, with warmer temperatures correlating with earlier nesting for both species. The number of nests laid each year has also increased annually by 12.4% for green turtles and 8.1% for loggerheads but did not have a significant relationship with any phenological measures (onset, peak, end, or duration of nesting season). Our data suggest that marine turtles in the Cayman Islands are shifting the onset of nesting toward cooler periods outside peak summer months however there was no change to the peak of nesting for either species. Whether this shift mitigates the impacts of rising temperatures on clutch incubation and offspring sex ratios remains unclear.

## Introduction

Under conditions of climate change, species survival depends on their capacity to adapt [1,2]. While short-lived species may evolve rapidly, longer-lived species often rely on phenotypic plasticity or behavioural shifts [3]. For example, many species adjust by altering geographic ranges [4,5] whilst others may change their phenology—the timing of life-cycle events [6]. Such phenological shifts are widespread across ecosystems in a wide range of organisms [7–10], with rising temperatures linked to changes in the onset and duration of reproductive events [11–14]. One example of species phenology shifting with rising temperatures is the nesting season of marine turtles [15–17].

Marine turtles are particularly vulnerable to climate change due to the linkage between the thermal environment and critical physiological processes, including temperature-sex-determination [18]. Temperature plays a key role in the development of marine turtle embryos, hatching success and hatchling sex ratios [19]. Higher temperatures lead to female-skewed populations and have also been shown to influence the nesting phenology at locations worldwide [15] (e.g., loggerhead turtles (*Caretta caretta*) in Florida, United States [20], North Cyprus [21] and Brazil [22]; leatherback turtles (*Dermochelys coriacea*) in St. Croix, US Virgin Islands [23]; green turtles (*Chelonia mydas*) in North Cyprus [17] and in Florida [20]). Temperature is also likely to affect reproduction and the phenology of breeding (e.g., through potential cues for migration timing and effects on food availability) and nesting, and a critical issue is whether phenological shifts enable species to adapt to a changing climate [24] by nesting under different environmental conditions [25,26]. The Foraging Site Hypothesis [27] suggests that marine turtles likely use environmental cues at their foraging areas to initiate migration. Nesting phenology may also be influenced by population size or structure [28]. In leatherback turtles in Costa Rica and green turtles in Cyprus, older and/or more experienced individuals tend to arrive earlier to nest and lay more clutches [17,25], and consequently, population demographics might drive earlier and longer lasting nesting seasons [29].

Despite their strong fidelity to nesting sites, marine turtles can adapt to thermal changes across scales – from wide-scale oceanic migrations shaped by regional oceanographic patterns [21], to selecting finer nearshore microhabitats amid fluctuating local temperatures [30] – potentially affecting their energy allocation. These could consequently influence courtship timing [31] and nesting behaviour. Thermal conditions along migratory pathways and in localised inter-nesting areas have the potential to contribute to variations in the timing and length of nesting seasons. The duration of the inter-nesting interval [32,33], the time between two consecutive nesting attempts within a nesting season [34] is also influenced by the thermal environment.

Temperature plays a fundamental role in shaping multiple life-history traits of marine turtles across all life stages. Their metabolic rates, growth rates, and overall energy acquisition are directly linked to the thermal environment [35]. Warmer conditions at foraging grounds can enhance somatic growth and reduce the time to sexual maturity [36], however, warmer oceans often result in diminished primary productivity and prey abundance, which can impose time-lagged effects on the breeding capacity of marine turtles in subsequent years [37]. In the reproductive phase, temperature is a key factor in egg maturation, clutch frequency, and overall fecundity. However, excessive heat stress could also reduce clutch size and egg viability, as prolonged exposure to high temperatures during follicle development may impair reproductive output [38]. These spatial and environmental complexities underscore the challenges in drawing broad conclusions about the effects of climate change on marine turtle nesting patterns from relatively short-term studies [39].

Given the importance of temperatures on marine turtle population dynamics, we examined the nesting phenology in a Caribbean rookery: the tropical beaches of the Cayman Islands, which represent important nesting grounds for loggerhead turtles and green turtles. These populations have both shown signs of recovery following a history of overexploitation followed by long-term conservation efforts by the local Department of Environment [40] and a head-start programme for green turtles by the Cayman Turtle Centre [41]. Despite the two species being morphologically similar, using similar nesting habitats, and displaying analogous migratory and nesting behaviours (as described by [42]), their distinct trophic levels – green turtles, generally feed two or three trophic levels lower in the food chain than loggerhead turtles [43] – suggest that their responses to climate change are unlikely to be the same [44]. In this study, we analysed the temporal nesting patterns of green and loggerhead marine turtles in relationship to sea surface temperature (SST) and extended the previous analysis on population trends by Blumenthal et al., (2021) to allow us to control for the effects of nesting magnitude on phenology. We hypothesise that higher temperatures will mean that turtles receive their cue to migrate earlier in the year and thus arrive earlier at their nesting beaches.

## Methods

### Nesting data

**Fig 1:**
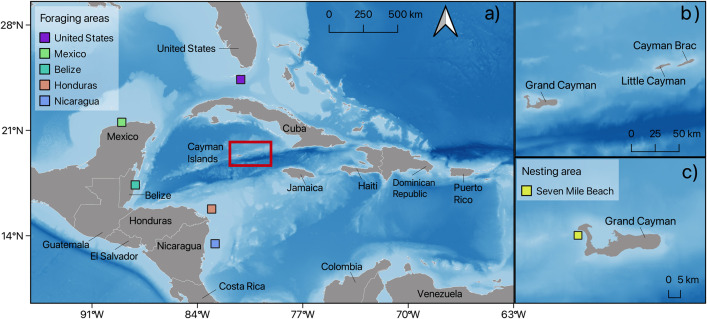
Map of study area and surrounding bathymetry (Global Bathymetric Chart of the Oceans (GEBCO) data) showing a) location of the Cayman Islands within the Caribbean Sea and foraging areas identified by previous satellite tracking [46], b) The three islands that comprise the Cayman Islands: Grand Cayman, Little Cayman and Cayman Brac, c) Grand Cayman showing the location of the sea surface temperature cell (SST) offshore from the nesting beach (Seven Mile Beach). Map produced using QGIS software. Administrative boundaries from OpenDataSoft World Administrative Boundaries dataset, licensed under OGL v3.0.

The Cayman Islands consist of three small, inhabited islands in the Caribbean Sea: Grand Cayman, Little Cayman, and Cayman Brac (Fig 1). Beaches were monitored during the loggerhead and green turtle nesting seasons (April to November) in Grand Cayman from 1999 to 2024 and in Little Cayman and Cayman Brac intermittently from 1998 to 2024, with standardised annual monitoring taking place in Little Cayman from 2014 to 2024 and Cayman Brac from 2012 to 2024 [40]. Following methods by [40,45], nesting beaches were surveyed on foot at least twice weekly throughout the nesting season, and many beaches monitored daily. Only confirmed nests, where clutches were located or signs of hatching recorded, were used in nest counts. Nest monitoring was not carried out in Little Cayman in 2020 due to Covid-19 travel restrictions. All fieldwork was conducted by the Cayman Islands Department of Environment (DoE), under the mandate of National Conservation Act section 6 (subsections (g – i)) to “monitor the populations of indigenous or migratory species and identify endangered, threatened, endemic or other species and their critical habitats requiring protection” and “for the purposes of research, collect or obtain samples” of protected species. All research was subject to ethical approval by the University of Exeter Ethics Committee (Application ID: 491773).

### Population trends

The trends in the annual number of green turtle nests across the three islands were analysed using generalized additive mixed models (GAMMs) with the ‘*gamm*’ function from the R package ‘*mgcv*’ [47]. A negative binomial distribution was applied to account for overdispersion in the nest counts (i.e., the observed variance in counts exceeding what would be expected under a Poisson distribution, often due to unmodeled heterogeneity such as variable environmental conditions or clustering of nesting events). To account for autocorrelation in the time series data, we tested several autoregressive moving average (ARMA) correlation structures using the corARMA function from the ‘*nlme*’ package [48]. The best-fitting model for each island was selected based on the lowest Akaike Information Criterion (AIC) value. The final models included a smooth term for year, with knot selection (k) adjusted for each location, and predictions were generated with associated confidence intervals. Model diagnostics were performed using autocorrelation (ACF) and partial autocorrelation (PACF) plots of the residuals to ensure model adequacy.

### Determining nesting phenology

We conducted separate phenological analyses for each species. Owing to the shorter time series and lower magnitude of nesting in Little Cayman and Cayman Brac, the phenological analysis was conducted for Grand Cayman only. Due to small sample sizes (number of nests = 1, 18 and 2, respectively), we excluded years 1999–2001 for green turtles. We described the overall nesting phenology using a set of equations based on time series of nest counts [49,50] analysed using the R package ‘*phenology*’ [51]. This model applies a negative-binomial distribution to each ordinal day and incorporates seven parameters to describe nesting phenology: (1) the date of peak nesting, (2) the average number of nests at the peak, (3) the season’s duration, (4) the minimum number of nests laid outside the nesting season, (5) the duration from the season’s start to the peak, (6) the duration from the peak to the end (with the start and end estimated via parameter 4), and (7) a negative-binomial parameter that accounts for dispersion around the mean. The model used all recorded nests to estimate the start and end dates of nesting seasons (parameters 1, 5, and 6), which provided a more accurate representation of nesting phenology than relying on the first and last nesting attempts recorded, which may be sporadic events that do not accurately reflect the underlying seasonal nesting phenology [52]. The analysis assumes that nesting does not plateau at the peak, consistent with the bell-shaped distribution of nest counts observed at the study sites. To minimise constraints on parameters governing nesting phenology (e.g., start, peak, and end), the maximum nesting rate for each season was initially estimated while holding “shape” parameters constant. The maximum value was then fixed, and the shape parameters were estimated in a subsequent step. Parameters were first estimated using maximum likelihood, followed by Bayesian MCMC with the Metropolis–Hastings algorithm and an adaptive proposal distribution over 10,000 iterations, assuming Gaussian priors for parameter distributions. An example of phenology for each year and species on the same island (Grand Cayman) is shown in S1 and S2 Figs. For the overall temporal distribution of nests, we considered the most recent five years of the data (2020–2024) to represent current nesting patterns. However, in Little Cayman, we used data from 2019–2024, excluding 2020 due to a lack of monitoring that year.

Additionally, for comparison between methods and to allow comparison to other studies that use only one method, we used our raw data to conduct the same phenology analysis: with onset of nesting each year determined as the 5th percentile of lay dates from clutches deposited at the rookery, while the end of the nesting season was defined as the 95th percentile of those dates. Annual metrics were calculated, including the median ordinal day of nesting and the duration of the nesting season, the latter being defined as the number of days between the 5^th^ and 95^th^ percentiles of clutches laid. Results for this analysis are presented in S2 File and Supporting Figures and Tables: S8–S10 Figs, S2 and S3 Tables. An example of phenology for each year and species on the same island (Grand Cayman) is shown in S3 and S4 Figs.

### Predictors of nesting phenology

Daily SST records were obtained from E.U. Copernicus Marine Service Information (https://doi.org/10.48670/moi-00173) and consisted of hourly measurements that were averaged to obtain a daily average temperature. They were spatially averaged using the ‘*terra*’ package [53] for five 0.25° x 0.25° grids: one adjacent to an important green and loggerhead turtle nesting beach in Grand Cayman (Seven Mile Beach) and four broadscale foraging areas, identified from satellite tracking of green turtles: north coast of Mexico, east coast of Belize (Belize/Guatemala feeding area), northeast Honduras, and southern Florida Keys (USA) and loggerhead turtles on the east coast of Nicaragua (Fig 1 and S5 Fig, [46]. We applied a sliding window approach using the R package ‘*climwin*’ [54] to identify the strongest predictor of nesting onset. The annual nesting onset dates were derived from the models using the R package ‘*phenology’* described above. The SST adjacent to the nesting beach between the 1^st^ March – 7^th^ April for loggerhead turtles and the 13^th^ April – 5^th^ May for green turtles was found to be the strongest predictor of nesting onset (see S1 File for details). Thus, we used the mean temperatures recorded between the mentioned dates as a predictor variable to estimate the effects of sea surface temperature.

We quantified trends in the (1) nesting onset, (2) peak nesting date, (3) end and (4) duration of nesting seasons by performing linear regressions (F-test) against year, SST and magnitude of nesting. To consider the system in an integrative manner, we used generalized linear models (GLMs) with a Gaussian distribution with responses (1) nesting onset, (2) peak nesting date, (3) end, and (4) duration of nesting seasons for each species and SST, total number of nests laid in each year (magnitude), and year as predictors. We applied backward stepwise deletion based on Akaike Information Criterion (AIC) to select the most parsimonious model, retaining only significant predictors (p < 0.05). To test our hypothesis that higher SST will mean that turtles receive their cue to migrate earlier in the year and thus arrive earlier at their nesting beaches, we used a correlation test. This test assessed whether temperatures in the months associated with when turtles are likely leaving their foraging habitats correlate with the timing of the nesting season.

## Results

### Population trends

**Fig 2:**
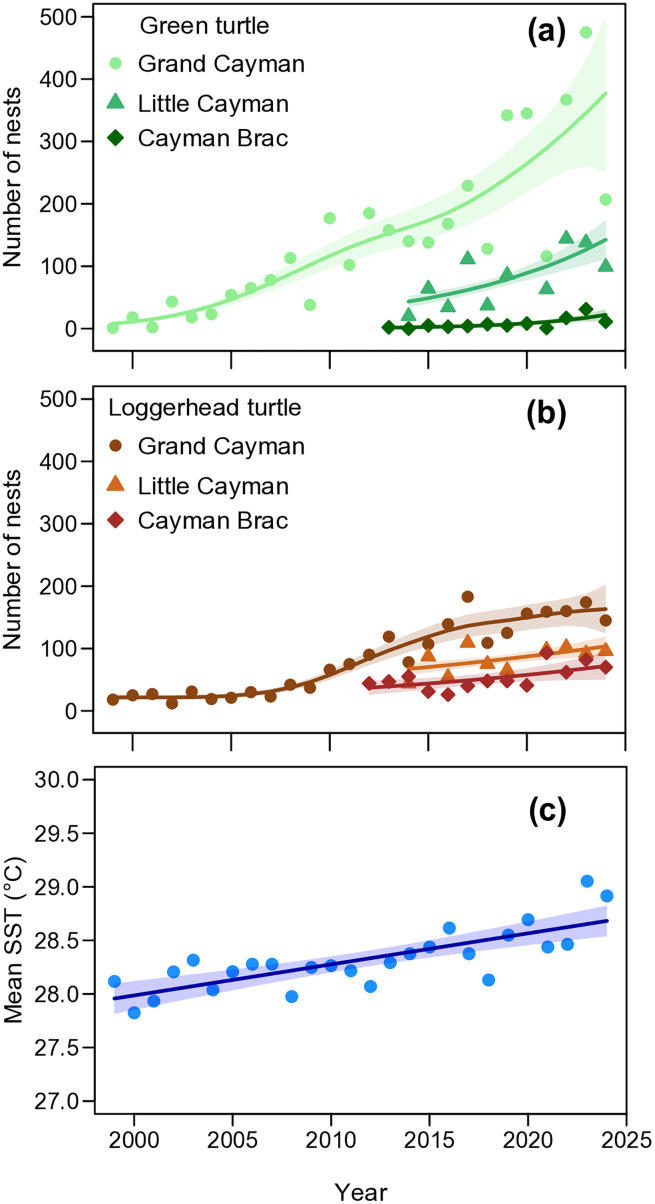
Annual number of green turtle (a) and loggerhead turtle (b) nests in the Cayman Islands, 1999−2024 (4670 green turtle clutches and 3696 loggerhead clutches in total). The annual number of nests in Grand Cayman is shown by filled dots, in Little Cayman by triangles and in Cayman Brac by diamonds. Solid curve: generalised additive model regression; shaded curves: approximate 0.95 pointwise confidence intervals. (c) Mean annual sea surface temperature (SST) recorded off the nesting beach in Grand Cayman, Cayman Islands, from 1999 to 2024. Each blue dot represents a mean annual SST measurement. The dark blue line indicates the overall temperature trend over time, fitted using a linear regression model. The grey shaded area represents the 95% confidence interval around the trendline.

We estimated that between 1999 and 2024, 4670 green turtle clutches and 3696 loggerhead turtle clutches were laid across the Cayman Islands. The GAM regressions in Fig 2 all showed a significant increasing trend in the annual number of nests for both species on all islands (S1 Table). The estimated mean annual rate of increase in nesting in Grand Cayman, based on the long-term dataset (1999–2024) was 12.4% for green turtles and 8.1% for loggerhead turtles. For the shorter period 2014–2024, when all three islands were monitored, the rates were 6.5% for green turtles and 2.6% for loggerhead turtles in Grand Cayman, 7.1% for green turtles and 2.1% for loggerhead turtles in Little Cayman, and 13.6% for green turtles and 5.7% for loggerhead turtles in Cayman Brac (Fig 2). Concurrently, the mean sea surface temperature (SST) has increased significantly over time (p < 0.01, R2 = 0.34; Fig 2c).

### Nesting phenology of green turtles

**Fig 3:**
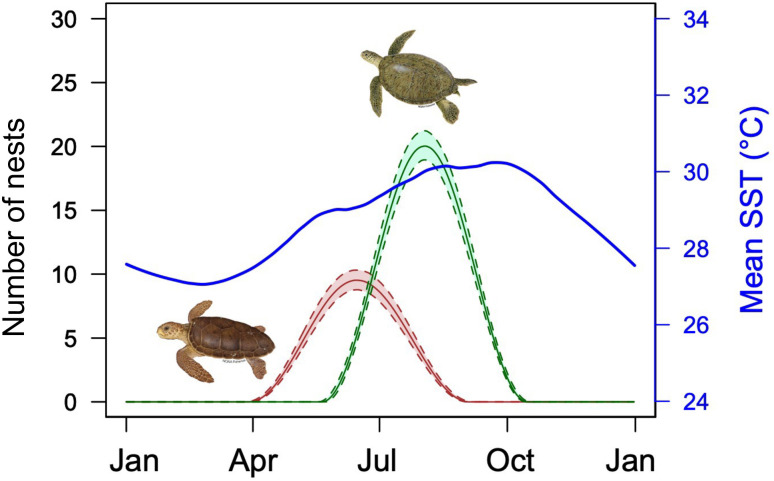
Phenology of green and loggerhead turtle nesting seasons in Grand Cayman from 2020-2024, based on nest counts. Solid lines: quantiles curve based on mcmc. Shaded area: credibility interval polygon based on MCMC. Green turtle shown in green and loggerhead turtle shown in brown. The trend in sea surface temperature during the same period is represented by the blue line.

**Fig 4:**
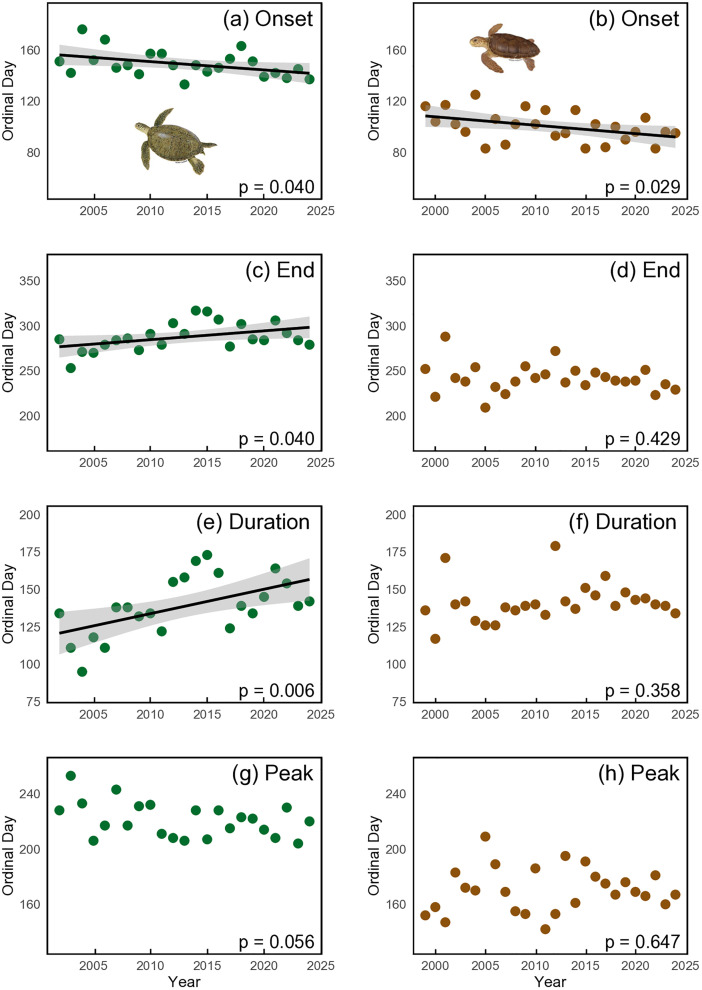
Phenology of green and loggerhead turtle nesting in Grand Cayman rookery. (a, b) nesting onset, (c, d) end of nesting season, (e, f) duration of nesting season and (g, h) peak day of nesting. Black lines in (a, b, e, f): linear regression according to season; shaded areas: 95% CI. Note that the trendline is shown only where linear regression was significant.

**Fig 5:**
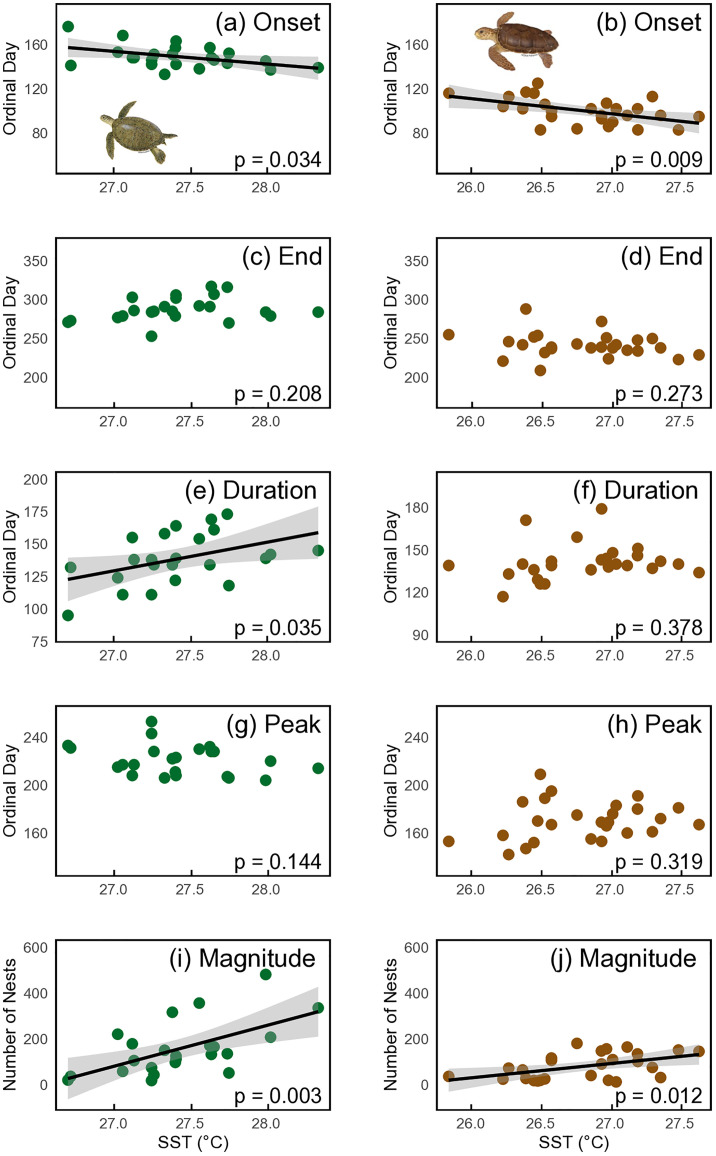
Relationship between the phenological parameters of green and loggerhead turtles nesting in the Grand Cayman rookery and sea surface temperature (SST). (a, b) nesting onset, (c, d) end of nesting season, (e, f) duration of nesting season, (g, h) peak day of nesting and (i, j) nesting magnitude. Black lines in (a, b, e, f): linear regression according to season; shaded areas: 95% CI. Note that the trendline is shown only where linear regression was significant.

Using the ‘*phenology*’ package, we found that the nesting season for green turtles in the Cayman Islands starts at the end of May, peaks in August and ends by mid-October (Fig 3 and S6 Fig). Over the past 23 years (2002−2024) there has been a significant advancement in the onset of nesting at a rate of 0.6 days.yr^-1^ (p = 0.04, R2 = 0.19). This rate of advancement represents a total shift of approximately 14 days earlier in the onset of the nesting season. Additionally, a significant trend was observed for the end of the nesting season, which has been delayed by an average of 1.0 days.yr^-1^ (p = 0.04, R2 = 0.19). Consequently, the overall duration of the nesting season increased significantly, extending by 1.6 days per year (p = 0.01, R2 = 0.31), while the day of peak nesting showed no significant change over the period (p = 0.06, R2 = 0.16, Fig 4 and Table 1). SST was significantly related to the onset of the nesting season (p = 0.04, R2 = 0.19), duration of the nesting season (p = 0.04, R2 = 0.19), and magnitude of nesting (p < 0.01, R2 = 0.35), indicating that warmer conditions are associated with earlier onsets, longer durations, and increased nesting activity (Fig 5). SST was not significantly related to the peak of nesting (p = 0.14, R2 = 0.10). No significant relationships were found between magnitude of nesting and duration, end, or onset of nesting (p > 0.05, Table 1 and S7 Fig).

**Table 1:** Summary of linear regression analysis between nesting parameters and SST for green (2002-2024) and loggerhead turtles (1999-2024) in Grand Cayman, Cayman Islands.

Pair	Green Turtle			Loggerhead		
	F	R^2^	p-value	F	R^2^	p-value
Year vs.						
Duration	9.52	0.31	**0.01**	0.88	0.04	0.36
End	4.79	0.19	**0.04**	0.65	0.03	0.43
Onset	4.77	0.19	**0.04**	5.36	0.18	**0.03**
Peak	4.09	0.16	0.06	0.22	0.01	0.65
SST vs.						
Magnitude	11.46	0.35	**<0.01**	10.40	0.30	**<0.01**
End	1.69	0.07	0.21	1.25	0.05	0.28
Onset	5.05	0.19	**0.04**	8.10	0.25	**0.01**
Duration	5.05	0.19	**0.04**	0.82	0.03	0.37
Peak	2.31	0.10	0.14	0.57	0.02	0.42
Magnitude vs.						
Duration	2.72	0.12	0.11	2.60	0.10	0.12
End	0.88	0.04	0.36	0.10	<0.01	0.76
Onset	2.98	0.12	0.10	5.48	0.19	**0.03**
Peak	2.89	0.12	0.10	0.14	0.01	**0.71**

GLMs with stepwise deletion revealed sea surface temperature (SST) significantly influenced nesting onset, but year and magnitude had no effect. For every 1°C increase in April SST, the onset of nesting advanced by 11.3 days. For nesting peak, no predictors were significant, including year, SST, or magnitude. Nesting duration increased significantly over time, while SST and magnitude were not significant. Similarly, nesting end occurred later over time, driven significantly by year, with no significant effects from SST or magnitude in the final model (Table 2).

**Table 2:** Summary of stepwise Generalized Linear Model (GLM) results for predictors of nesting phenology in green and loggerhead turtles in Grand Cayman, Cayman Islands.

		Year		SST		Magnitude	
Species	Model	t-value	p-value	t-value	p-value	t-value	p-value
Green turtle	Onset	−0.95	0.35	−2.25	**0.04**	0.25	0.81
	Peak	−2.02	0.06	−1.52	0.14	−0.08	0.94
	Duration	3.09	**0.01**	1.10	0.29	−1.23	0.23
	End	2.19	**0.04**	1.30	0.21	−1.27	0.22
Loggerhead turtle	Onset	0.11	0.92	−2.85	**0.01**	−0.56	0.58
	Peak	0.12	0.91	0.76	0.46	−0.13	0.90
	Duration	−1.40	0.18	0.91	0.37	1.92	0.68
	End	−1.07	0.30	−1.12	0.28	1.12	0.28

### Nesting phenology of loggerhead turtles

For loggerhead turtles, using the ‘*phenology*’ package, we found that the nesting season starts at the beginning of April, peaks in mid-June and ends by the end of August (Fig 3 and S6 Fig). Over the 26-year period (1999−2024), the onset of nesting has significantly advanced at a rate of 0.7 days.yr^-1^ (p = 0.03, R2 = 0.18). This rate of advancement represents a total shift of approximately 18 days earlier in the onset of the nesting season. No significant trends were observed for the end of the nesting season (p = 0.43, R2 = 0.03), the duration of the nesting season (p = 0.36, R2 = 0.04) or the peak of nesting over the period (p = 0.65, R2 = 0.01, Fig 4 and Table 1). SST was significantly related to the onset of the nesting season (p = 0.01, R2 = 0.25) and magnitude of nesting (p < 0.01, R2 = 0.30), indicating that warmer conditions are associated with earlier onsets and increased nesting activity (Fig 5), but SST was not significantly related to the peak of nesting (p = 0.46, R2 = 0.02). Additionally, a significant relationship was found between magnitude and onset of nesting (p = 0.03, R2 = 0.19) with larger nesting years starting earlier. No significant relationships were found between SST and duration or end of the nesting season, or between magnitude and duration or end of nesting (p > 0.05, Table 1 and S7 Fig).

GLMs with stepwise deletion revealed sea surface temperature (SST) significantly influenced nesting onset, but year and magnitude had no effect. For every 1°C increase in March SST, the onset of nesting advanced by 13.4 days. For nesting peak, no predictors were significant, including year, or magnitude. Similarly, nesting duration was not significantly affected by magnitude, year, or SST. Nesting end also showed no significant effects of SST, magnitude, or year (Table 2).

## Discussion

For marine turtles, shifts in phenology driven by warming conditions may represent the most effective short-term response to cope with climate change [52]. We observed that both marine turtle species nesting in Grand Cayman are advancing the onset of nesting, but only green turtles are increasing the duration of their nesting season, indicating interspecific variation in marine turtle response to increasing temperatures. In addition, we did not observe any shift in the peak nesting season, raising questions about what is driving earlier nesting. We discuss our key findings and provide recommendations for advancing this research to better understand the extent of population recovery, resilience and adaptive potential in response to climate change.

### Nesting onset

After accounting for population growth, our analysis revealed significant advancements in the onset of nesting for both species. While some marine turtle populations show shifting nesting peaks [17,21], our study found no such associations. This discrepancy may be related to factors such as latitude and season length, which could influence phenological responses differently in our study region compared to other populations. Notably, the onset of nesting showed a strong negative correlation with SST, reinforcing its role as a key environmental driver of the start of the nesting season. The significant increase in the duration of the nesting season for green turtles could allow this species to maintain similar overall thermal conditions for embryonic development considering the predicted changes in temperature. Marine turtles in many regions predominantly nest during the warmest months of the year, typically aligning with summer or peak seasonal temperatures [52]. An extended nesting season could enable green turtles to experience a broader range of thermal conditions, potentially buffering against extreme temperatures. If nesting is spread out over a longer period, some clutches may still develop under cooler conditions even if peak-season temperatures are rising, which could help mitigate the impacts of climate change on embryonic development.

### Length of nesting season

While both species appeared to exhibit phenological shifts in response to SST, differences in their responses suggest species-specific adaptations. Green turtles demonstrated a greater extension of the nesting season compared to loggerhead turtles, potentially reflecting differences in their reproductive physiologies or resource use. Additionally, green turtles exhibited a greater increase in nesting over the study period, both in terms of magnitude and rate of increase which in turn may drive increased variation in arrival dates among females. The relationship between SST and nesting numbers was also much stronger in green turtles, suggesting that their reproductive patterns may be more closely linked to temperature fluctuations. For instance, higher SSTs have been associated with shorter inter-nesting intervals, which may enable green turtles to lay more clutches per season [32,55]. These findings are consistent with studies from other regions, such as in Australia, where earlier nesters exhibited higher clutch frequencies [38]. Loggerhead turtles, by contrast, displayed an advance in the onset of the nesting season by approximately 18 days over the study period, but with no significant increase in nesting duration or shift in the end of the season, despite earlier arrivals. This pattern could reflect their lower plasticity in reproductive timing overall, as well as distinct environmental drivers acting on different phenological parameters: Monsinjon et al. [22] found that the onset of nesting in loggerheads is primarily triggered by temperature cues at key foraging sites, whereas nesting duration is more closely tied to population size, allowing for independent responses to SST and demographic factors. As primary consumers feeding on seagrass, which is more directly impacted by environmental fluctuations, green turtles may exhibit earlier responses, such as initiating migration to reproductive areas in response to these changes.

### Consistency with previous studies

Our findings are consistent with studies exploring phenological responses of marine turtles to climate change. Monsinjon et al. [56] found that loggerhead turtles in the Mediterranean exhibit significant advancements in nesting onset driven by rising SSTs, suggesting that earlier nesting may mitigate thermal stress on clutches, though it could increase exposure to suboptimal conditions later in the season. This aligns with our observed advancements in nesting onset for both green (0.6 days.yr ⁻ ¹) and loggerhead turtles (0.7 days.yr ⁻ ¹) in the Cayman Islands, although the lack of a shift in peak nesting dates indicates that thermal cues possibly primarily influence the initiation of nesting. Similarly, [16] demonstrated that phenological shifts, such as earlier nesting, may help marine turtles maintain their thermal niche in a warming climate, but these adjustments may be limited in tropical regions where seasonal temperature variations are less pronounced. This supports our observation that, despite earlier nesting onsets and an extended nesting season for green turtles (1.6 days.yr⁻¹), the absence of significant peak nesting shifts in the Cayman Islands suggests that these populations may not fully counteract the impacts of rising temperatures on clutch incubation and sex ratios. These studies underscore the critical role of SST in driving phenological responses and highlight the challenges tropical populations face in adapting to rapid climate warming.

### Role of sea surface temperature in nesting phenology

The sliding window analysis identified SST offshore from the nesting site as the most critical predictor of nesting onset for both species. While SSTs at the foraging areas were also significant, the stronger correlations observed for SSTs recorded weeks prior to nesting suggest a combination of influences on migration and reproductive timing. This supports the Foraging Site Hypothesis [27], indicating that marine turtles likely use environmental cues from their foraging areas to initiate migration, however conditions closer to the nesting site may further regulate the final stages of reproductive readiness and nesting. This underscores the importance of sea surface temperatures, often used as a proxy for the thermal environment experienced by turtles, as both a migratory cue and a physiological regulator, influencing the timing of nesting in ectothermic species such as marine turtles. However, as different individuals and species may forage or rest at various depths, future studies should explore how well surface temperatures correlate with the temperatures turtles encounter subsurface. Weishampel et al. [20] predicted that increases in Caribbean SSTs would result in earlier nesting and a change in duration of the nesting seasons of both green and loggerhead turtles, which, in part, aligns with our results, particularly for green turtles. In fact, SSTs in the Caribbean have been increasing over the past century, with studies reporting rises of 0.5 °C to 1 °C [57]. Our data, showing a 1°C increase from 1999 to 2024, suggest a relatively rapid warming trend in our study period. This warming trend has significant implications for marine ecosystems, including coral reefs and marine turtle nesting sites, which are highly sensitive to temperature changes.

### Influence of analytical methods on phenological trends

The method chosen for the phenological analysis influences both the results and the interpretation of phenological trends. The ‘*phenology’* package, with its Bayesian model tailored for seasonal studies, provides biologically meaningful estimates of onset, peak, and end dates and captures uncertainty through credible intervals. In contrast, raw data percentiles describe general trends but may miss key phenological patterns. For green turtles, the ‘*phenology’* package identified significant trends in the onset (advancing 0.6 days.yr^-1^), end (delaying 1.0 days.yr^-1^), and duration (extending 1.6 days.yr^-1^) of nesting seasons. In contrast, despite detecting the same advancement in the onset of nesting (0.6 days.yr^-1^), the raw data approach did not detect a significant trend for the end of the nesting season, with the increase in nesting season duration slightly shorter (0.8 days.yr^-1^). For loggerhead turtles, the ‘*phenology*’ package detected a 0.7 days.yr^-1^ advancement in onset, while the raw data showed a slower rate (0.4 days.yr^-1^) and a significant increase in nesting season duration (0.6 days.yr^-1^), which the ‘*phenology*’ package did not detect. Overall, in this study, the ‘*phenology*’ package better captured long-term phenological shifts, smoothed through noise, and reduced the risk of overfitting compared to the raw data approach. However, when high-quality count data are available, the necessity of the ‘*phenology*’ package’s modelled estimates versus the reliability of raw observations warrants consideration, raising questions about whether smoothed estimates or actual data best reflect true phenological patterns.

### Population recovery and demographic shifts

The significant increase in nesting activity observed for both green and loggerhead turtles in the Cayman Islands aligns with previous reports of population recovery in these species at this site [40] and may reflect successful conservation efforts, such as egg and female protection, which have helped to mitigate historical exploitation [40]. A captive breeding operation contributed to the increase in the Grand Cayman green turtle population [58,59], and loggerhead turtle populations began to increase following the cessation of a legal traditional turtle fishery in 2008, as they were never captive bred. Globally, green marine turtle populations are increasing, many after centuries of direct take [60–62]. Rising temperatures may further accelerate population recovery by increasing the speed of maturation and egg production. While many sites are reporting higher numbers of nesting females, a concurrent reduction in adult body size has been observed [63,64]. This raises questions about whether this reflects the recruitment of a larger number of smaller, younger females into the population or a genuine reduction in size at sexual maturity, which could have implications for their reproductive output and long-term population viability. Such demographic changes could drive variation in nesting phenology, as individual-level plasticity in response to environmental cues, such as advancing nesting dates with rising sea surface temperatures, and population-level shifts, like increasing proportions of less experienced breeders, influence timing and reproductive output [17]. For instance, younger turtles typically nest later in the season [25], so a decreasing mean age of nesters could counteract temperature-driven advancements and help explain the lack of a significant shift in mean peak nesting dates observed in our study. Similar demographic influences on phenology have been documented in leatherback turtles, where multidecadal trends in nesting timing were linked to changes in population age structure [23]. Investigating such individual variation in our population would provide valuable insights, however long-term individual-based data are not yet available.

### Conservation implications

Our findings have significant conservation implications, particularly in the context of climate change. As SSTs continue to rise [57], if earlier nesting and longer seasons continue to occur, this will influence hatchling survival and sex ratios. Given the temperature-dependent sex determination in marine turtles and the thermal conditions needed for successful embryonic development, earlier nesting may be adaptive, offering cooler temperatures for some clutches than would be experienced otherwise. Our data suggest that marine turtles in the Cayman Islands may not be shifting nesting to a degree that will mitigate the impact of rising temperatures on clutch incubation and offspring sex ratios, particularly in the tropics where seasonal shifts are not as rapid as at higher latitudes [65] and turtles could show limited phenological responses to warming trends. This highlights the need to understand how individual females respond to rising temperatures in their timing of breeding [17] or whether they produce more clutches to compensate for thermal stress. Irrespective, conservation strategies should prioritise climate adaptation measures, such as protecting nesting beaches, particularly if they may be more male producing than others, and retaining natural vegetation cover at nesting sites [24].

### Future research directions

This study represents a critical step in understanding phenological shifts of marine turtles in the Caribbean and underscores the need for long-term, multi-site studies that utilise standardised methodologies to disentangle site-specific factors from global trends. Investigating the interplay between environmental cues at foraging and nesting sites will provide deeper insights into the mechanisms driving these phenological shifts. Furthermore, exploring the broader ecological consequences of these shifts, such as changes in food availability, hatchling sex ratios and nesting success, will enhance our understanding of marine turtle resilience and adaptive capacity in a warming world.

## Supporting information

S1 File Methods. (DOCX)

S2 File Results. (DOCX)

S1 Fig Seasonality of green sea turtle *Chelonia mydas* nesting activity measured in Grand Cayman in the Cayman Islands during 2002–2024, using the ‘*phenology*’ package. Central plain line is the fitted Bayesian model, and dashed lines represent the 95% credible interval of the seasonality of nesting.
(DOCX)

S2 Fig Seasonality of loggerhead sea turtle *Caretta caretta* nesting activity measured in Grand Cayman in the Cayman Islands during 2002–2024, using the ‘*phenology*’ package. Central plain line is the fitted Bayesian model and dashed lines represent the 95% credible interval of the seasonality of nesting.
(DOCX)

S3 Fig Seasonality of green sea turtle (*Chelonia mydas*) nesting activity measured in Grand Cayman in the Cayman Islands during 2002–2024, using the raw data. The solid dark green line represents the fitted Generalized Additive Model (GAM) estimating the seasonal trend in nesting activity. Black dots indicate the actual daily nest counts recorded throughout the year. The shaded light green area shows the 95% confidence interval around the GAM fit, while the dashed dark green lines mark the upper and lower boundaries of this confidence interval.
(DOCX)

S4 Fig Seasonality of loggerhead sea turtle (*Caretta caretta*) nesting activity measured in Grand Cayman in the Cayman Islands during 1999–2024, using raw data. The solid brown line represents the fitted Generalized Additive Model (GAM) estimating the seasonal trend in nesting activity. Black dots indicate the actual daily nest counts recorded throughout the year. The shaded light brown area shows the 95% confidence interval around the GAM fit, while the dashed brown lines mark the upper and lower boundaries of this confidence interval.
(DOCX)

S5 Fig Foraging and nesting areas for green and loggerhead turtles used for Climwin analysis shown in detail. Foraging areas were previously identified from satellite tracking study (Blumenthal et al. 2006). Map produced using QGIS software. Administrative boundaries from OpenDataSoft World Administrative Boundaries dataset, licensed under OGL v3.0. Surrounding bathymetry from Global Bathymetric Chart of the Oceans (GEBCO) data.
(DOCX)

S6 Fig Dynamics of green and loggerhead turtle nesting seasons, in Grand Cayman and Cayman Brac between 2020–2024, and in Little Cayman between 2019–2023, based on nest counts. Solid lines: quantiles curve based on MCMC. Shaded area: credibility interval polygon based on MCMC.
(DOCX)

S7 Fig Relationships between nesting season parameters for *Chelonia mydas* (Cm) and *Caretta caretta* (Cc), using the *phenology* package. The plots illustrate the following correlations: (1) total number of nests (magnitude) vs. duration, (2) total number of nests (magnitude) vs. onset, (3) total number of nests (magnitude) vs. end, and (4) total number of nests (magnitude) vs year. Solid lines represent linear regression fits, and text annotations display the Pearson correlation coefficients (r). Shaded areas indicate the 95% confidence interval (CI). Note that trendlines are shown only where linear regression was statistically significant. Titles above the plots specify the relationship depicted in each panel.
(DOCX)

S8 Fig Phenology of green and loggerhead turtle nesting in Grand Cayman, Cayman Islands rookery, using raw data. (a, b) 5^th^ percentile day of annual nesting onset, (c, d) 95^th^ percentile day of annual nesting, (e, f) Duration of nesting season and (g, h) Annual median day of nesting. Black lines in (a, b, e, f): linear regression according to season; shaded areas: 95% CI. Note that the trendline is shown only where linear regression was significant.
(DOCX)

S9 Fig Relationship between phenology parameters of green and loggerhead turtle nesting in Grand Cayman, Cayman Islands rookery, and sea surface temperature (SST), using raw data. (a, b) 5^th^ percentile day of annual nesting onset, (c, d) 95^th^ percentile day of annual nesting, (e, f) Duration of nesting season, (g, h) Annual median day of nesting and (i, j) Magnitude of nesting. Black lines in (a, b, e, f): linear regression according to season; shaded areas: 95% CI. Note that the trendline is shown only where linear regression was significant.
(DOCX)

S10 Fig Relationships between nesting season parameters using raw data for *Chelonia mydas* (Cm) and *Caretta caretta* (Cc). The plots illustrate the following correlations: (1) total number of nests (magnitude) vs. duration, (2) total number of nests (magnitude) vs. onset, (3) total number of nests (magnitude) vs. end, and (4) total number of nests (magnitude) vs year. Solid lines represent linear regression fits, and text annotations display the Pearson correlation coefficients (r). Shaded areas indicate the 95% confidence interval (CI). Note that trendlines are shown only where linear regression was statistically significant. Titles above the plots specify the relationship depicted in each panel.
(DOCX)

S1 Table Summary of generalised additive model (GAM) analysis. The table shows the significance of smooth terms in explaining trends in annual nest counts for green turtles and loggerhead turtles across the Cayman Islands: Grand Cayman, Little Cayman, and Cayman Brac.
(DOCX)

S2 Table Summary of linear regression analysis of nesting season parameters against year using raw data for green (2002–2024) and loggerhead turtles (1999–2024) in Grand Cayman, Cayman Islands. (DOCX)

S3 Table Summary of stepwise Generalized Linear Model (GLM) results for predictors of nesting phenology in green and loggerhead turtles in Grand Cayman, Cayman Islands, using raw data. (DOCX)
