# Supplementary material for: Phenology of nesting marine turtles in the Cayman Islands
Source: PLoS One. 2025 Dec 31;20(12):e0338445. doi: 10.1371/journal.pone.0338445 (PMC12782257; doi:10.1371/journal.pone.0338445)
Supplement: S1 File — (DOCX) [file pone.0338445.s001.docx]

**S1 Methods**

SST offshore from the nesting site was identified by the sliding window analysis to be the strongest predictor of nesting onset for both green and loggerhead turtles, with SST at Seven Mile Beach the most important variable for both species. For green turtles, the best-fitting linear model had an AICc value of -8.15 and a significant p-value of 0.03. The model slope (ModelBeta) was -9.17 (SE = 2.57), indicating that higher temperatures led to earlier nesting. The most critical period of SST influence spanned 63 to 40 days before the onset of nesting, corresponding to April 13th to May 5th. For loggerhead turtles, the best-fitting linear model had an AICc value of -7.71 and a highly significant p-value of <0.001. The model slope (ModelBeta) was -11.59 (SE = 3.33), also indicating a negative relationship between SST and the timing of nesting. The critical window for SST influence was between 64 and 26 days before the onset of nesting, corresponding to March 1st to April 7th. Linear and quadratic models were tested, and top models were identified based on ΔAICc values. Randomisation analysis was performed to assess the significance of the climate windows.
