# Supplementary material for: Phenology of nesting marine turtles in the Cayman Islands
Source: PLoS One. 2025 Dec 31;20(12):e0338445. doi: 10.1371/journal.pone.0338445 (PMC12782257; doi:10.1371/journal.pone.0338445)
Supplement: S2 File — (DOCX) [file pone.0338445.s002.docx]

**S1 results**

For green turtles, considering the raw data (5^th^ and 95^th^ percentile of nesting distribution), there has been a significant advancement in the onset of nesting at an average rate of 0.6 days.yr^-1^. No significant trend was observed for the end of the nesting season. However, the overall duration of the nesting season increased significantly, extending by 0.8 days.yr^-1^, while the median day of nesting showed no significant change over the period (S8 Fig and S2 Table). SST was significantly related to the onset of nesting (p = 0.02, R² = 0.24) and magnitude of nesting (p < 0.01, R² = 0.35), indicating that warmer conditions are associated with earlier onsets and increased nesting activity. SST was not significantly related to the median date of nesting (p = 0.21, R² = 0.07), to duration (p = 0.06, R² = 0.07) or end of nesting season (p = 0.79, R² < 0.01, S9 Fig). Additionally, a significant relationship was found between magnitude and onset of nesting (p = 0.03, R² = 0.199), suggesting that earlier onsets are associated with higher nesting magnitude. No significant relationships were found between magnitude and duration or end of nesting (p > 0.05, S10 Fig and S2 Table).

Linear models with stepwise deletion revealed that sea surface temperature (SST) significantly influenced nesting onset, with a 1°C increase in April SST advancing nesting by 10.3 days. Neither year nor magnitude had significant effects on onset. For the median date of nesting, no predictors were significant, including year, SST, or magnitude. Nesting duration increased significantly over time, with no significant effects from SST or magnitude. Nesting end showed no significant predictors, with all variables removed in the final stepwise model, indicating no clear drivers for the end of the nesting season (S3 Table).

For loggerhead turtles using the raw data (5^th^ and 95^th^ percentiles of nesting), over the 26-year period (1999-2024), the onset of nesting has significantly advanced at a rate of 0.4 days.yr-1. No significant trend was observed for the end of the nesting season, while the duration of the nesting season has increased significantly by an average of 0.6 days.yr-1. The median date of nesting showed no significant trend over the period (S8 Fig and S2 Table). SST was significantly related to the onset of nesting (p = 0.03, R² = 0.19) and magnitude of nesting (p < 0.01, R² = 0.30), indicating that warmer conditions are associated with earlier onsets and increased nesting activity. SST was not significantly related to median nesting date (p = 0.26, R² = 0.05) or to duration (p = 0.19, R² = 0.07) or end of nesting season (p = 0.70, R² = 0.01, S9 Fig) Significant relationships were also found between magnitude and duration (p < 0.01, R² = 0.26) and magnitude and onset of nesting (p = 0.02, R² = 0.21), suggesting that earlier onsets and longer durations are associated with higher nesting magnitude. No significant relationships were found between SST and duration or end of the nesting season, or between magnitude and end of nesting (p > 0.05, S10 Fig and S2 Table).

Linear models with stepwise deletion showed that nesting onset was significantly influenced by magnitude, with no significant effects from SST or year. Median nesting date showed no significant predictors, including year, SST, or magnitude. Nesting duration increased significantly with magnitude, while SST and year were not significant. Nesting end showed no significant predictors, with all variables removed in the final stepwise model, suggesting no clear drivers for the end of the nesting season (S3 Table).
