# Supplementary material for: Phenology of nesting marine turtles in the Cayman Islands
Source: PLoS One. 2025 Dec 31;20(12):e0338445. doi: 10.1371/journal.pone.0338445 (PMC12782257; doi:10.1371/journal.pone.0338445)
Supplement: S1 Fig — Central plain line is the fitted Bayesian model, and dashed lines represent the 95% credible interval of the seasonality of nesting. (DOCX) [file pone.0338445.s003.docx]

**S1 Fig.** **Seasonality of green sea turtle *Chelonia mydas* nesting activity measured in Grand Cayman in the Cayman Islands during 2002-2024, using the ‘*phenology*’ package.** Central plain line is the fitted Bayesian model, and dashed lines represent the 95% credible interval of the seasonality of nesting.
