# Supplementary material for: Phenology of nesting marine turtles in the Cayman Islands
Source: PLoS One. 2025 Dec 31;20(12):e0338445. doi: 10.1371/journal.pone.0338445 (PMC12782257; doi:10.1371/journal.pone.0338445)
Supplement: S3 Fig — The solid dark green line represents the fitted Generalized Additive Model (GAM) estimating the seasonal trend in nesting activity. Black dots indicate the actual daily nest counts recorded throughout the year. The shaded light green area shows the 95% confidence interval around the GAM fit, while the dashed dark green lines mark the upper and lower boundaries of this confidence interval. (DOCX) [file pone.0338445.s005.docx]

**S3 Fig.** **Seasonality of green sea turtle (*Chelonia mydas*) nesting activity measured in Grand Cayman in the Cayman Islands during 2002–2024, using the raw data.** The solid dark green line represents the fitted Generalized Additive Model (GAM) estimating the seasonal trend in nesting activity. Black dots indicate the actual daily nest counts recorded throughout the year. The shaded light green area shows the 95% confidence interval around the GAM fit, while the dashed dark green lines mark the upper and lower boundaries of this confidence interval.
