# Supplementary material for: Phenology of nesting marine turtles in the Cayman Islands
Source: PLoS One. 2025 Dec 31;20(12):e0338445. doi: 10.1371/journal.pone.0338445 (PMC12782257; doi:10.1371/journal.pone.0338445)
Supplement: S4 Fig — The solid brown line represents the fitted Generalized Additive Model (GAM) estimating the seasonal trend in nesting activity. Black dots indicate the actual daily nest counts recorded throughout the year. The shaded light brown area shows the 95% confidence interval around the GAM fit, while the dashed brown lines mark the upper and lower boundaries of this confidence interval. (DOCX) [file pone.0338445.s006.docx]

**S4 Fig.** **Seasonality of loggerhead sea turtle (*Caretta caretta*) nesting activity measured in Grand Cayman in the Cayman Islands during 1999–2024, using raw data.** The solid brown line represents the fitted Generalized Additive Model (GAM) estimating the seasonal trend in nesting activity. Black dots indicate the actual daily nest counts recorded throughout the year. The shaded light brown area shows the 95% confidence interval around the GAM fit, while the dashed brown lines mark the upper and lower boundaries of this confidence interval.
