# Supplementary material for: Phenology of nesting marine turtles in the Cayman Islands
Source: PLoS One. 2025 Dec 31;20(12):e0338445. doi: 10.1371/journal.pone.0338445 (PMC12782257; doi:10.1371/journal.pone.0338445)
Supplement: S5 Fig — Foraging areas were previously identified from satellite tracking study (Blumenthal et al. 2006). Map produced using QGIS software. Administrative boundaries from OpenDataSoft World Administrative Boundaries dataset, licensed under OGL v3.0. Surrounding bathymetry from Global Bathymetric Chart of the Oceans (GEBCO) data. (DOCX) [file pone.0338445.s007.docx]

**S5 Fig.** **Foraging and nesting areas for green and loggerhead turtles used for Climwin analysis shown in detail.** Foraging areas were previously identified from satellite tracking study (Blumenthal et al. 2006). Map produced using QGIS software. Administrative boundaries from OpenDataSoft World Administrative Boundaries dataset, licensed under OGL v3.0. Surrounding bathymetry from Global Bathymetric Chart of the Oceans (GEBCO) data.
