# Supplementary material for: Phenology of nesting marine turtles in the Cayman Islands
Source: PLoS One. 2025 Dec 31;20(12):e0338445. doi: 10.1371/journal.pone.0338445 (PMC12782257; doi:10.1371/journal.pone.0338445)

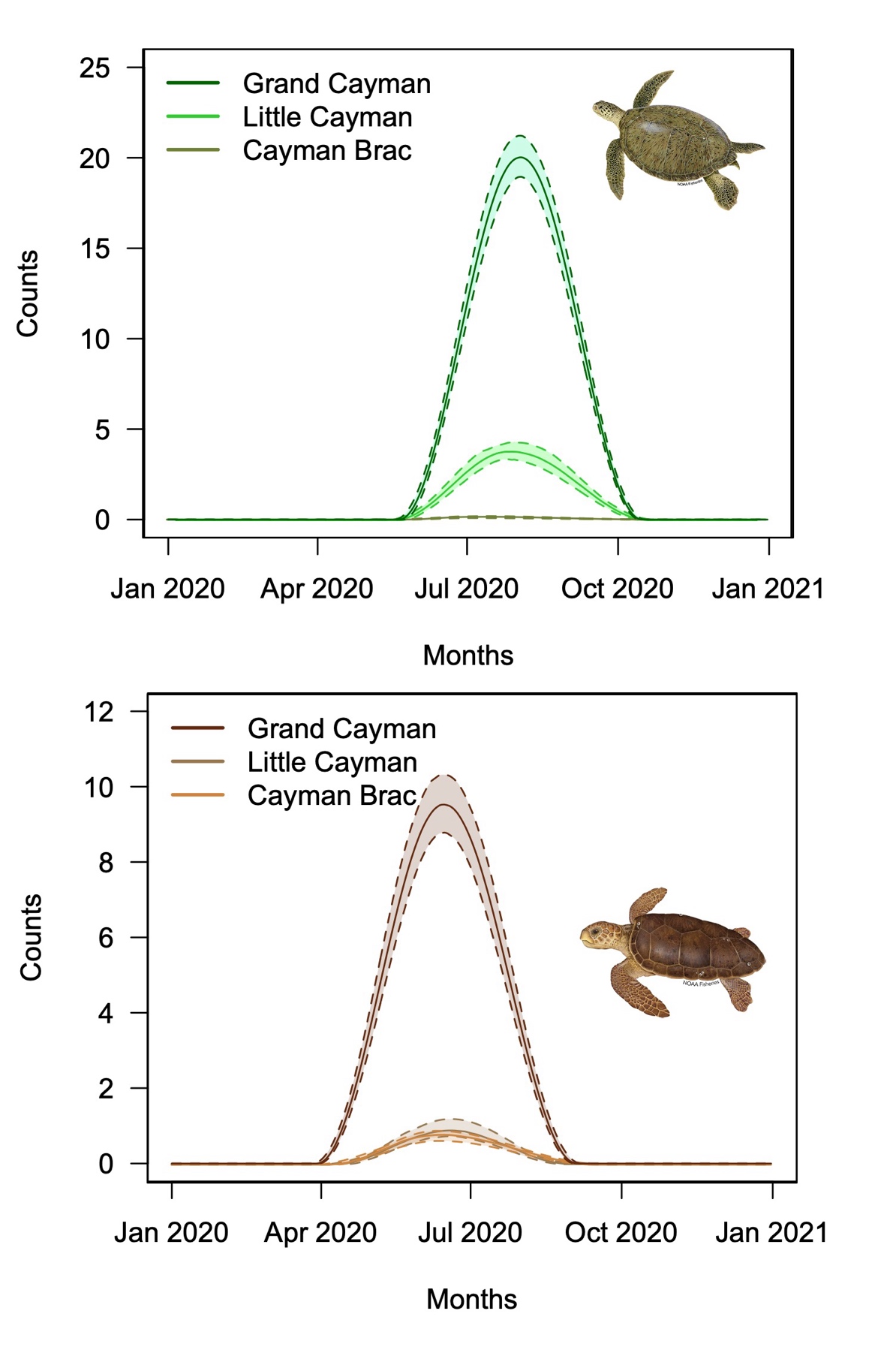


**S6 Fig.** **Dynamics of green and loggerhead turtle nesting seasons, in Grand Cayman and Cayman Brac between 2020-2024, and in Little Cayman between 2019-2023, based on nest counts.** Solid lines: quantiles curve based on MCMC. Shaded area: credibility interval polygon based on MCMC.


Supplement: S6 Fig — Solid lines: quantiles curve based on MCMC. Shaded area: credibility interval polygon based on MCMC. (DOCX) [file pone.0338445.s008.docx]
