# Supplementary material for: Phenology of nesting marine turtles in the Cayman Islands
Source: PLoS One. 2025 Dec 31;20(12):e0338445. doi: 10.1371/journal.pone.0338445 (PMC12782257; doi:10.1371/journal.pone.0338445)
Supplement: S9 Fig — (a, b) 5th percentile day of annual nesting onset, (c, d) 95th percentile day of annual nesting, (e, f) Duration of nesting season, (g, h) Annual median day of nesting and (i, j) Magnitude of nesting. Black lines in (a, b, e, f): linear regression according to season; shaded areas: 95% CI. Note that the trendline is shown only where linear regression was significant. (DOCX) [file pone.0338445.s011.docx]

**S9 Fig.** **Relationship between phenology parameters of green and loggerhead turtle nesting in Grand Cayman, Cayman Islands rookery, and sea surface temperature (SST), using raw data.** (a, b) 5^th^ percentile day of annual nesting onset, (c, d) 95^th^ percentile day of annual nesting, (e, f) Duration of nesting season, (g, h) Annual median day of nesting and (i, j) Magnitude of nesting. Black lines in (a, b, e, f): linear regression according to season; shaded areas: 95% CI. Note that the trendline is shown only where linear regression was significant.
