# Supplementary material for: Phenology of nesting marine turtles in the Cayman Islands
Source: PLoS One. 2025 Dec 31;20(12):e0338445. doi: 10.1371/journal.pone.0338445 (PMC12782257; doi:10.1371/journal.pone.0338445)
Supplement: S10 Fig — The plots illustrate the following correlations: (1) total number of nests (magnitude) vs. duration, (2) total number of nests (magnitude) vs. onset, (3) total number of nests (magnitude) vs. end, and (4) total number of nests (magnitude) vs year. Solid lines represent linear regression fits, and text annotations display the Pearson correlation coefficients (r). Shaded areas indicate the 95% confidence interval (CI). Note that trendlines are shown only where linear regression was statistically significant. Titles above the plots specify the relationship depicted in each panel. (DOCX) [file pone.0338445.s012.docx]

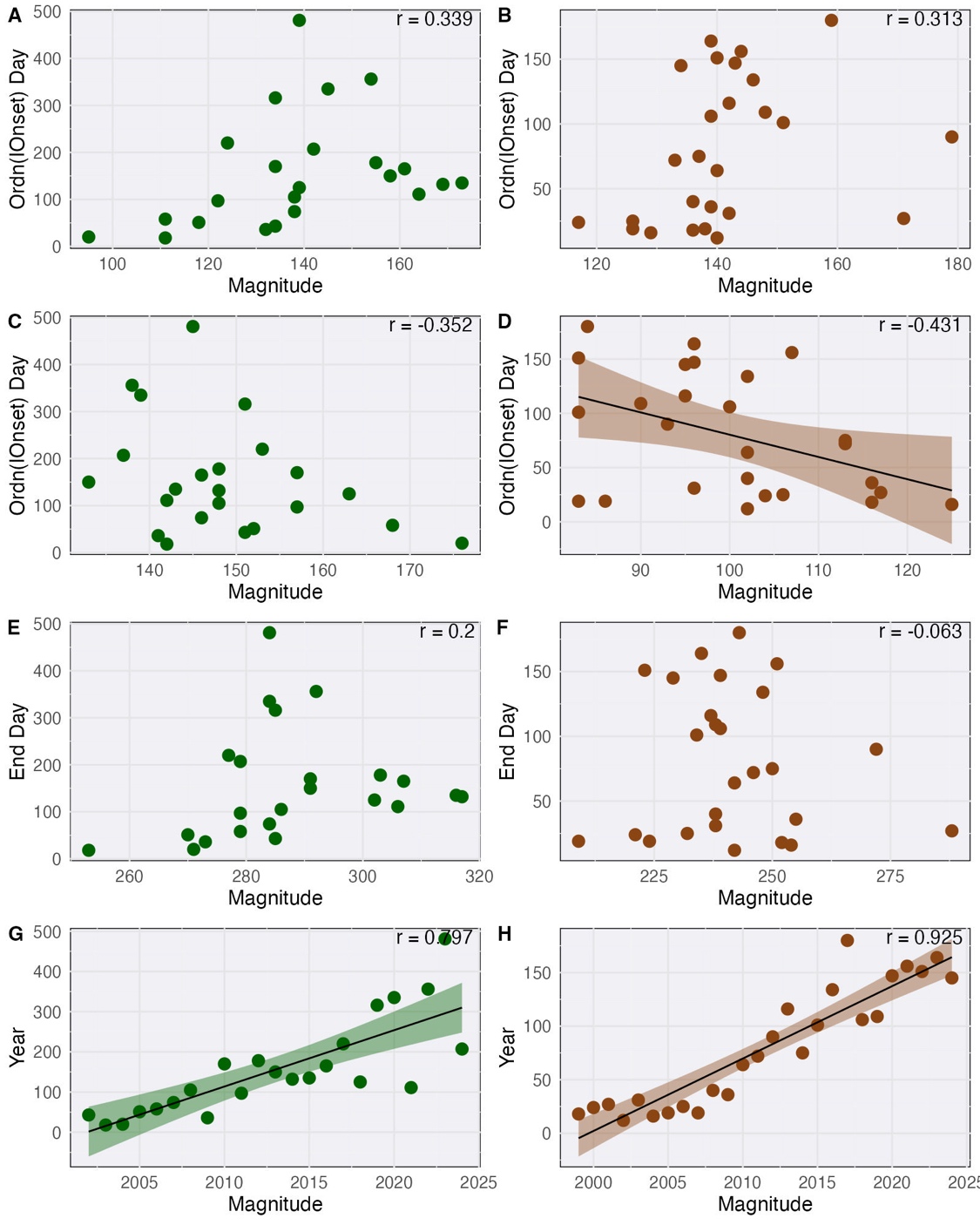


**S10 Fig.** **Relationships between nesting season parameters using raw data for *Chelonia mydas* (Cm) and *Caretta caretta* (Cc).** The plots illustrate the following correlations: (1) total number of nests (magnitude) vs. duration, (2) total number of nests (magnitude) vs. onset, (3) total number of nests (magnitude) vs. end, and (4) total number of nests (magnitude) vs year. Solid lines represent linear regression fits, and text annotations display the Pearson correlation coefficients (r). Shaded areas indicate the 95% confidence interval (CI). Note that trendlines are shown only where linear regression was statistically significant. Titles above the plots specify the relationship depicted in each panel.
