# Supplementary material for: Phenology of nesting marine turtles in the Cayman Islands
Source: PLoS One. 2025 Dec 31;20(12):e0338445. doi: 10.1371/journal.pone.0338445 (PMC12782257; doi:10.1371/journal.pone.0338445)
Supplement: S1 Table — The table shows the significance of smooth terms in explaining trends in annual nest counts for green turtles and loggerhead turtles across the Cayman Islands: Grand Cayman, Little Cayman, and Cayman Brac. (DOCX) [file pone.0338445.s013.docx]

**S1 Table.** **Summary of generalised additive model (GAM) analysis.** The table shows the significance of smooth terms in explaining trends in annual nest counts for green turtles and loggerhead turtles across the Cayman Islands: Grand Cayman, Little Cayman, and Cayman Brac.

| Species | Location | F-value | EDF (Estimated Degrees of Freedom) | p-value |
| --- | --- | --- | --- | --- |
| Green turtles | Grand Cayman | 144.69 | 3.77 | < 0.01 |
|  | Little Cayman | 42.39 | 1 | < 0.01 |
|  | Cayman Brac | 48.16 | 1 | < 0.01 |
| Loggerhead turtles | Grand Cayman | 147.98 | 5.15 | < 0.01 |
|  | Little Cayman | 11.85 | 1 | < 0.01 |
|  | Cayman Brac | 6.35 | 1 | 0.03 |
