# Supplementary material for: Phenology of nesting marine turtles in the Cayman Islands
Source: PLoS One. 2025 Dec 31;20(12):e0338445. doi: 10.1371/journal.pone.0338445 (PMC12782257; doi:10.1371/journal.pone.0338445)
Supplement: S2 Table — (DOCX) [file pone.0338445.s014.docx]

**S2 Table.** **Summary of linear regression analysis of nesting season parameters against year using raw data for green (2002-2024) and loggerhead turtles (1999-2024) in Grand Cayman, Cayman Islands.**

| **Pair** | **Green Turtle** | | | **Loggerhead** | | |
| --- | --- | --- | --- | --- | --- | --- |
|  | **F** | **R^2^** | **p-value** | **F** | **R^2^** | **p-value** |
| Year vs. |  |  |  |  |  |  |
| duration | 5.93 | 0.22 | **0.02** | 5.76 | 0.19 | **0.02** |
| end | 0.56 | 0.03 | 0.46 | 0.35 | 0.01 | 0.56 |
| onset | 6.30 | 0.23 | **0.02** | 6.20 | 0.21 | **0.02** |
| median nesting | 1.52 | 0.07 | 0.23 | 0.19 | 0.01 | 0.67 |
| SST vs. |  |  |  |  |  |  |
| magnitude | 11.46 | 0.35 | **<0.01** | 10.40 | 0.30 | **<0.01** |
| end | 0.07 | <0.01 | 0.79 | 0.16 | 0.01 | 0.70 |
| onset | 6.67 | 0.24 | **0.02** | 5.76 | 0.19 | **0.03** |
| duration | 3.89 | 0.16 | 0.06 | 1.84 | 0.07 | 0.19 |
| median nesting | 1.68 | 0.07 | 0.21 | 1.33 | 0.05 | 0.26 |
| Magnitude vs.  duration | 3.21 | 0.13 | 0.09 | 8.41 | 0.26 | **0.09** |
| end | 0.07 | <0.01 | 0.79 | 1.00 | 0.04 | 0.33 |
| onset | 5.21 | 0.20 | **0.03** | 6.41 | 0.21 | **0.02** |
| median nesting | 1.93 | 0.08 | 0.18 | 0.10 | <0.01 | 0.75 |
