# Supplementary material for: Phenology of nesting marine turtles in the Cayman Islands
Source: PLoS One. 2025 Dec 31;20(12):e0338445. doi: 10.1371/journal.pone.0338445 (PMC12782257; doi:10.1371/journal.pone.0338445)
Supplement: S3 Table — (DOCX) [file pone.0338445.s015.docx]

**S3 Table. Summary of stepwise Generalized Linear Model (GLM) results for predictors of nesting phenology in green and loggerhead turtles in Grand Cayman, Cayman Islands, using raw data.**

|  |  | Year | |  | SST | |  | Magnitude | |
| --- | --- | --- | --- | --- | --- | --- | --- | --- | --- |
| Species | Model | t-value | p-value |  | t-value | p-value |  | t-value | p-value |
| Green turtle | Onset | -0.780 | 0.445 |  | -2.582 | **0.017** |  | -0.183 | 0.857 |
|  | Median | -0.100 | 0.922 |  | -0.534 | 0.600 |  | -0.462 | 0.650 |
|  | Duration | 2.435 | **0.024** |  | 0.811 | 0.427 |  | -0.296 | 0.953 |
|  | End | 0.830 | 0.417 |  | -0.06 | 0.953 |  | -0.473 | 0.641 |
| Loggerhead turtle | Onset | -0.051 | 0.960 |  | -1.156 | **0.260** |  | -2.531 | **0.018** |
|  | Median | -0.018 | 0.986 |  | -1.063 | 0.299 |  | 0.176 | 0.862 |
|  | Duration | -0.442 | 0.663 |  | 0.048 | 0.962 |  | 2.9 | **0.008** |
|  | End | -0.535 | 0.598 |  | -0.922 | 0.367 |  | 1.135 | 0.268 |
